# Mercurial Transudation

**Published:** 1835

**Authors:** B. F. Williams

**Affiliations:** Student of Medicine, late of Sandusky, Ohio; Cincinnati


### XIII.—Mercurial Transudation.

*A case of Mercurial Transudation from the cutaneous surface, under the use of the pilulæ hydrargyri.* Communicated by B. F. Williams, Student of Medicine, late of Sandusky, Ohio.

Mrs. A. C., aged 36, of strumous constitution, became affected with induration of one of her breasts, while nursing her third or fourth child. She was ordered a milk and farinaceous diet, with topical applications, and the daily use of the *blue pill.* Pursuing this course for several months, she found herself better. She was then siezed with ague and fever, for which she took the sulphate of quinine, which excited copious perspiration. In wiping the perspiration from her face she observed on her hand and on her handkerchief a great number of minute globules of fluid mercury. On making this discovery, and observing the phenomenon on successive days, she discontinued the use of the pill, and soon after the mercury ceased to exude. It was distinctly and repeatedly seen by numerous persons. She assured me that she had no unusual feelings or indisposition during the employment of the medicine.

*Cincinnati, June,* 1835.

